# Association Between Peripheral Vascular Endothelial Function and Progression of Open-Angle Glaucoma

**DOI:** 10.1097/MD.0000000000003055

**Published:** 2016-03-11

**Authors:** Chun-Hsiu Liu, Wei-Wen Su, Shian-Sen Shie, Shih-Tsung Cheng, Cheng-Wen Su, Wang-Jing Ho

**Affiliations:** From the Department of Ophthalmology(C-HL, W-WS); Department of Internal medicine (S-SS), Chang Gung Memorial Hospital, Chang Gung University College of Medicine, Taoyuan; Department of Cardiology (S-TC, C-WS), Buddhist Tzu Chi General Hospital Taipei Branch, Xindian, New Taipei City; Department of Cardiology (W-JH), Chang Gung Memorial Hospital, Chang Gung University College of Medicine, Taoyuan, Taiwan.

## Abstract

The aim of the study is to evaluate the relationship between Humphrey visual field progression and peripheral vascular endothelial function in patients with open-angle glaucoma (OAG), assessed by noninvasive endothelium-dependent flow-mediated vasodilation (FMD).

Forty OAG patients, among which 22 had normal-tension glaucoma (NTG) and 18 had primary open-angle glaucoma (POAG) were enrolled. Each enrolled patient underwent a thorough ophthalmological examination including the Humphrey visual field test and measurement of FMD via high-resolution 2-dimensional ultrasonographic imaging of the brachial artery. Blood samples were evaluated for biochemistry and lipid profiles as well as levels of high-sensitivity C-reactive protein (hsCRP). The annual change of threshold sensitivity of the visual field in each test location were analyzed with pointwise linear regression. The correlation between long-term visual field progression and FMD was evaluated.

A mean follow-up of 7.47 ± 1.84 years revealed a faster progression rate over the superior visual field in all 40 OAG patients (superior field −0.24 ± 0.67 dB/y, inferior field −0.10 ± 0.59 dB/y, *P* = 0.37). However, only the annual sensitivity change of the inferior peripheral field showed correlation with baseline FMD. There was no significant difference in the change slope of visual field between NTG and POAG patients.

A correlation between baseline brachial artery FMD and visual field progression was observed in the inferior peripheral field in patients with NTG and POAG. This result suggests that peripheral vascular endothelial dysfunction may be related to glaucoma progression.

## INTRODUCTION

Glaucoma is one of the leading causes of blindness worldwide. Glaucoma refers to a group of conditions typically characterized by a loss of retinal ganglion cells (RGC) with changes to the retinal nerve fiber layer and optic nerve head (ONH), which manifest themselves as a particular pattern of visual field loss. Traditionally, intraocular pressure (IOP) is considered the most important and the only modifiable risk factor for glaucomatous damage. The presence of normal-tension glaucoma (NTG), however, challenges the long-held pathophysiological concept of glaucoma based only on IOP. One hypothesis proposes that vascular factors contribute to NTG.^1^ For example, Harrington reported that patients with impaired ocular blood flow were more susceptible to glaucomatous damage despite normal IOPs.^2^ A higher prevalence of migraine headache, Raynaud phenomenon, ischemic vascular diseases, and sleep apnea in patients with NTG is also suggestive of a vascular origin in glaucoma pathogenesis.^3^

The vascular endothelium–smooth muscle complex is a complicated organ that maintains vascular homeostasis and regulates vascular tone via autocrine, paracrine, and endocrine properties. Endothelial dysfunction is considered an important factor for developing systemic diseases such as atherosclerosis, hypertension, and heart failure.^4^ Several reports have identified compromised vascular endothelial cell function in glaucoma patients.^5–9^ The vascular endothelial function can be assessed in several ways, and 1 well-established noninvasive method uses ultrasound to measure endothelium-dependent flow-mediated vasodilation (FMD) in brachial artery.^10^ This technique involves creating transient brachial arterial occlusion by cuff inflation followed by a sudden deflation, upon which a brief high-flow state is induced, which in turn increases shear stress of the brachial artery wall and triggers endothelial nitric oxide (NO) release and vasodilation.^11^ FMD is judged as the vascular response to NO and can be quantified as an index of vasomotor function.^4,11^ We previously reported that patients with open-angle glaucoma (OAG) had impaired FMD, and the impairment was greater in those with NTG than with POAG.^7,9^ The extent to which this dysfunction contributes to the glaucomatous progress is unknown, however. The purpose of the present study is to investigate the relationship between long-term visual field progression and vascular endothelial dysfunction measured by FMD in brachial artery.

## PATIENTS AND METHODS

### Participants

Forty patients with OAG (22 NTG and 18 POAG) were recruited from the glaucoma clinic of Chang Gung Memorial Hospital for this study. The diagnostic criteria for POAG were as follows: pretreatment IOP exceeded 21 mm Hg, open anterior chamber angles on gonioscopy, glaucomatous optic disc cupping (cup-to-disc ratio > 0.7 with thinning or notching of the neural rim), and typical glaucomatous visual field loss on Humphrey perimetry using the 30-2 Swedish interactive threshold algorithm (SITA) standard program (Carl Zeiss Meditec, Dublin, CA), which were reproducible in 2 consecutive tests. The diagnostic criteria for NTG were identical to those for POAG except that the untreated IOP never exceeded 21 mm Hg measured at varying intervals from 8 AM to 5 PM. Eyes with previous ocular trauma, ocular surgery, or ocular diseases other than glaucoma were excluded. We also excluded patients with systemic diseases, for example, diabetes mellitus, hypertension, congestive heart failure, hypercholesterolemia, cerebral vascular accident (CVA), or autoimmune diseases. Thus, none of our patients were taking systemic medications such as antihypertensive drugs, cholesterol-lowering agents, aspirin, or nitrates, and none were receiving hormone replacement therapy. The study followed the tenets of the Declaration of Helsinki and was approved by the Institutional Review Board of Chang Gung Memorial Hospital. All participants provided both verbal and written informed consent.

### Visual Field Evaluation

Visual field tests of the Humphrey 30-2 SITA standard program were performed at regular intervals every 6 months. The enrolled patients should have at least 5 reliable tests for progression analysis, each defined as < 20% fixation losses, < 33% false-positive errors, and < 33% false-negative errors. The raw data of retinal sensitivity (in dB) of the left eyes were exported for analyses. After excluding edge points (except the 2 nasal-most points across the horizontal midline) and the 2 points corresponding to the blind spot, threshold sensitivity of 52 test points were analyzed with pointwise linear regression.

### Biochemical Measurement

All participants received blood tests for biochemical profiles. Venous blood samples were collected after 12 hours of overnight fasting and processed for the following tests: fasting blood glucose, serum cholesterol, triglyceride, creatinine, alanine aminotransferase (ALT), uric acid, and the high-sensitivity C-reactive protein (hsCRP) levels.

### Measurement of Vascular Endothelial Function

All participants were referred to the vascular laboratory for the vascular ultrasound study after fasting for at least 8 hours. The brachial artery FMD was measured by high-resolution 2-dimensional (2-D) ultrasonic imaging as previously described.^11^ Briefly, using an Acuson L7 7.5 MHz linear array transducer on an Acuson Aspen ultrasound system (Acuson, Mountain View, CA), 2D images of the left brachial artery and pulsed Doppler flow velocity signals were obtained. Imaging was performed in a dimly lit and quiet room at a temperature of 22° to 25° C. Before the first scan, patients were rested in the supine position for at least 10 minutes and remained supine until the final recording was completed with electrocardiogram continuous monitoring. Blood pressure was measured from the right arm before imaging. Images were obtained 3 to 5 cm above the antecubital fossa. First, baseline 2D images were acquired, followed by pulsed Doppler blood flow velocity. To induce hyperemia, we used a 5.6-inch wide blood pressure cuff inflated to 250 mm Hg at the forearm to occlude the arterial blood flow completely. The arterial occlusion was maintained for 5 minutes before the cuff was deflated rapidly. At the same time, the transducer was carefully placed in an identical position. Pulsed Doppler velocity signals were then recorded at 5 to 10 seconds immediately after the deflation. At 60 seconds after cuff deflation, 2D images of the brachial artery were recorded for 5 seconds. To determine the endothelium-independent, nitroglycerin-mediated vasodilation (NMD), the patients were rested for another 10 minutes, followed by sublingual nitroglycerin spray (400 μg). Repeated brachial artery scans were performed at the same position 4 minutes after nitroglycerin administration.

### Image Analysis

Brachial artery diameter was measured by 2 observers who were blinded to clinical data of the patients. Special care was taken to ensure analyzing identical segments by identifying anatomic landmarks. Measurements were obtained from the anterior to the posterior M line (interface between media and adventitia of vessel wall) at the end diastole coinciding with the R-wave on the electrocardiogram. The diameter was calculated as the average of 3 end-diastolic frames. The FMD and NMD were calculated as the percentage change in brachial artery diameter in response to hyperemia and to nitroglycerin respectively:

FMD = [(VD_hyperemia_–VD_baseline_)/VD_baseline_] × 100%
NMD = [(VD_nitroglycerin_–VD_baseline_)/VD_baseline_] × 100%
VD = vessel diameter

### Statistical Analysis

Data were expressed as mean ± SD for continuous variables and percentage for categorical variables. The computational statistical environment R (http://www.r-project.org) was used to carry out large-scale pointwise linear regression analyses. For subtypes of glaucoma, the Mann–Whitney test was used to compare group means and the chi-square test for categorical data. For relationship between FMD and visual field progression on each test location, the Spearman correlation coefficient was calculated. A *P* value <0.05 was considered statistically significant.

## RESULTS

A total of 40 eyes from 40 individuals were included in the analyses: 18 were POAG and 22 were NTG. Table 1 lists the demographic data of both groups. There was no significant difference in age, gender distribution, initial mean deviation (MD), on Humphrey visual field, and biochemistry profile between these 2 groups. The FMD showed significant impairment in the NTG group (*P* = 0.017).

**TABLE 1:**
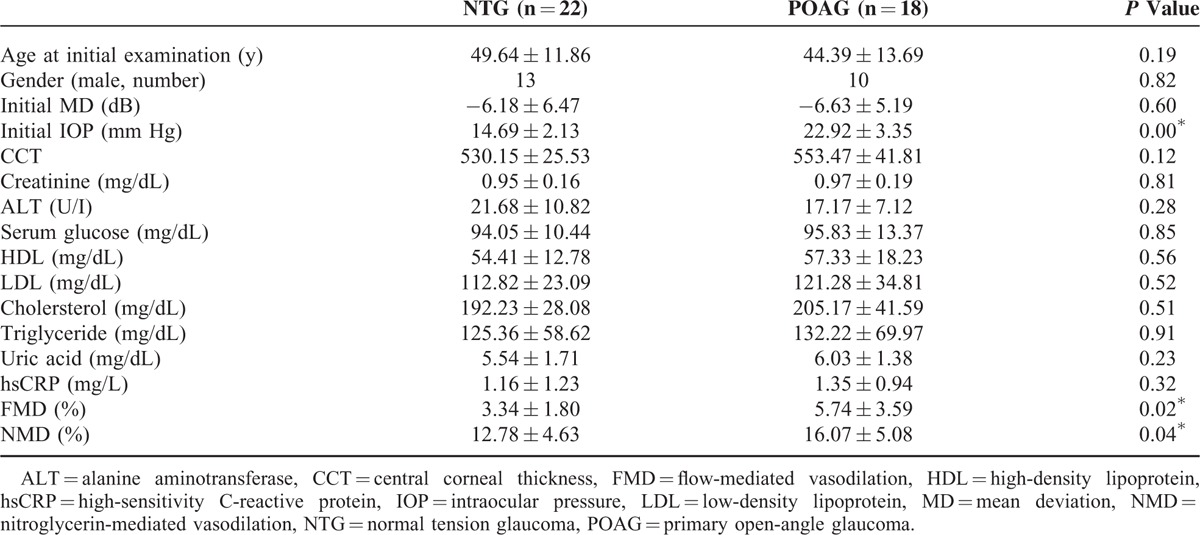
Baseline Characteristics in Patients With Normal-Tension Glaucoma and Primary Open-Angle Glaucoma

**FIGURE 1:**
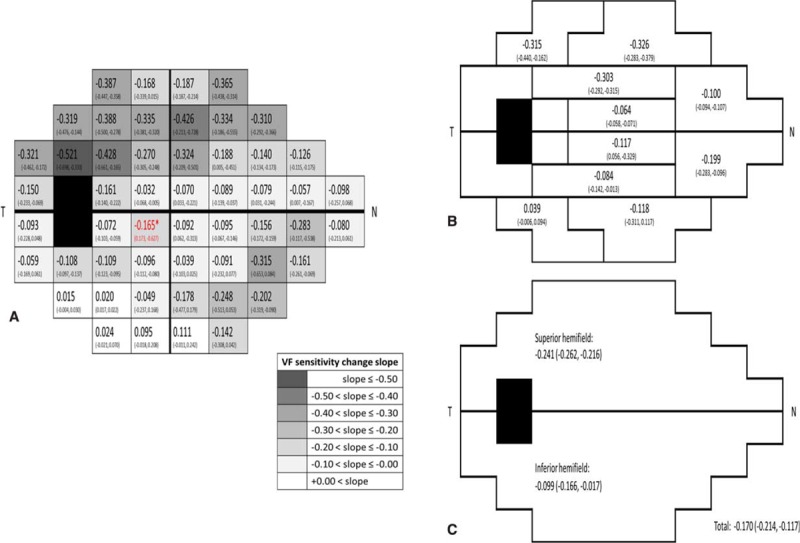
The average annual change of visual field sensitivity (dB/y) of OAG (NTG, POAG) for each test location (A), glaucoma hemifield test (GHT) zone (B), and hemifield (C), respectively. GHT = glaucoma hemifield test, N = nasal, NTG = normal-tension glaucoma, OAG = open-angle glaucoma, POAG = primary open-angle glaucoma, T = temporal.

Figure 1 shows the average annual change of visual field sensitivity for each test location, glaucoma hemifield test (GHT) zone, and hemifield, respectively. The progression is faster in the NTG group than in the POAG group, though statistically insignificant: NTG −0.21 ± 0.64 dB/y, POAG −0.14 ± 0.38 dB/y, *P* = 0.92. The superior hemifield progresses faster than the inferior hemifield: superior hemifield −0.24 ± 0.67 dB/y, inferior hemifield −0.10 ± 0.59 dB/y, but statistically insignificant: *P* = 0.37.

**FIGURE 2:**
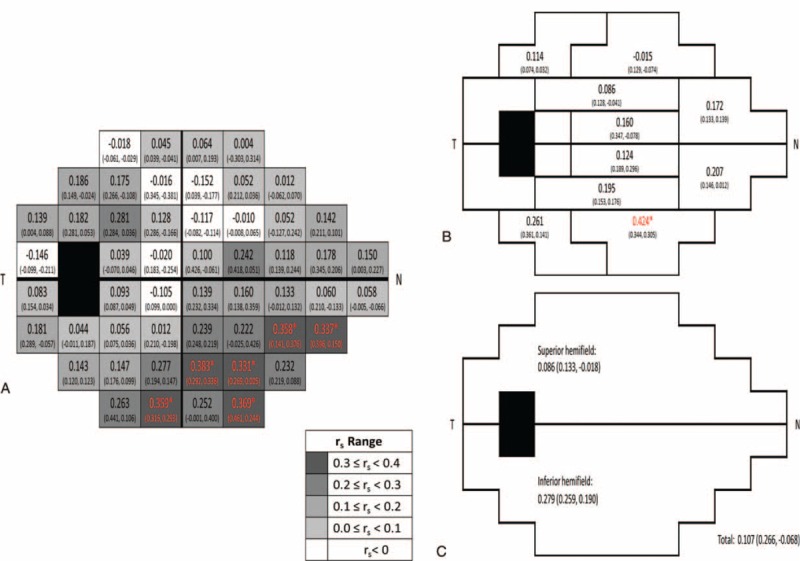
The correlation between visual field sensitivity change slope and FMD of OAG (NTG, POAG) for each test location (A), glaucoma hemifield test (GHT) zone (B), and hemifield (C), respectively. The *r*_*s*_ ranges are shown in the bottom right in grayscale. Red (^∗^) indicates the correlation was significant (*P* < 0.05). FMD = flow-mediated vasodilation, GHT = glaucoma hemifield test, N = nasal, NTG = normal-tension glaucoma, OAG = open-angle glaucoma, POAG = primary open-angle glaucoma, T = temporal.

Figure 2 shows the correlation between FMD and the average annual change of visual field sensitivity for each test location, GHT zone, and hemifield, respectively. Significant correlation was observed in 6 inferior peripheral test locations or GHT zone 9, approximating an inferior peripheral arcuate pattern.

## DISCUSSION

The present study demonstrates that FMD is correlated with visual field progression in the inferior peripheral area. To our knowledge, this is the first study to describe a relationship between peripheral vascular endothelial dysfunction and long-term visual field changes in patients with NTG and POAG.

Detection of functional progression plays a central role in the treatment of glaucoma. Albeit at present the Zeiss Humphrey Field Analyser remains the method of choice for monitoring functional changes, there is still a lack of consensus on the standard criterion for glaucoma progression assessment. Several algorithms have been proposed to identify visual field progression over time and they are either event-based or trend-based. In event-based analysis, the difference between baseline and follow-up examinations is compared to the test–retest variability from a separate sample of patients. When the observed changes exceed the predefined test–retest limit, progression was flagged based on a chosen cut-off range.^12^ One such examples is the commercially available guided progression analysis (GPA) software from the Humphrey Field Analyser. In contrast, a trend-based analysis detects temporal progression over serial measurements based on linear regression. The trend-based analysis can be a very powerful tool especially when numerous observations are available, and subtle changes can be spotted early on. The Humphrey Field Analyser also provides software for trend-based assessments of temporal progression for the mean deviation (MD) and the visual field index (VFI). Both progressive changes in MD and VFI represent a global evaluation of visual function, and localized defect in early glaucoma may be neglected. In this study, we evaluated the threshold sensitivity at each test location in the visual field by detecting changes over time using massive pointwise linear regression (PLR). We also assessed regional changes of the visual field according to the GHT clusters, in order to correlate zonal changes with vascular factors.

Alterations in ocular blood flow have been found to correlate with structural and functional changes in OAG.^13–18^ For example, Zink et al reported that lower blood volume in the inferior temporal neuroretinal rim is associated with greater corrected pattern standard deviation (CPSD) in standard automated perimetry (SAP).^19^ Using optical coherence tomography angiography, Jia et al showed that the disc flow index is reduced remarkably in glaucoma and is highly correlated with pattern standard deviation (PSD) of the visual field.^20^ Deokule et al reported that although the parapapillary blood flow does not correlate with visual field loss, the variance in mean parapapillary blood flow is significantly larger in OAG with visual field loss, suggestive of vascular abnormality.^21^ Both CPSD and PSD measure global changes of the visual field, and the correlation between vascular factors and focal visual field changes cannot be established using either of these measurements. In this study we used PLR to assess regional variation by measuring temporal change at each test location. The FMD represents a global vascular endothelial function and is considered as an index of vasomotor response for vital organs. Similar to earlier reports, our data showed a faster progression in the superior field,^22–24^ but only progression in the inferior peripheral field was correlated with FMD. Whether this finding implies that the superior peripheral retinal territory is more susceptible to vasomotor dysregulation remains to be elucidated. Second, our patients were relatively early glaucomas (MD = −6.18 ± 6.47 in the NTG and −6.63 ± 5.19 dB in the POAG group), their temporal changes of visual field loss might not be significant enough to show any difference in the early stage. As glaucoma progresses slowly, a longer follow-up is needed for further verification.

To sum up, our study demonstrates that the brachial artery FMD is significantly correlated with inferior peripheral visual field progression, suggesting there is a regional difference in the visual field in terms of vulnerability to vascular dysregulation.
